# Person references, change in footing, and agency positioning in psychotherapeutic conversations

**DOI:** 10.3389/fpsyg.2023.1206327

**Published:** 2023-06-30

**Authors:** Jarl Wahlström

**Affiliations:** Department of Psychology, University of Jyväskylä, Jyväskylä, Finland

**Keywords:** psychotherapy, conversation analysis, person reference, footing, agency positioning

## Abstract

This study contributes to the research on agency positioning in psychotherapy by looking at how clients and therapists, when discussing the client's difficulties, made use of two specific conversational practices, i.e., different grammatical forms of person reference and changes in footing, and what the consequences of this were for how the clients were positioned in relation to their problematic experiences. A data corpus of the first sessions of nine psychotherapies at a university training clinic in Finland was utilized. The uses of person references and changes in footing in therapists' initiative turns, clients' responses, and therapists' third position (recipient) actions were examined. The analysis showed that in initiative turns therapists usually used the second-person singular, as an invitation for the client to respond from his/her personal point of view, thus ascribing active agency to the client. When telling their problematic experiences, clients typically used so-called zero-person constructions, presenting such experiences as common to people in general, thus lessening their agency and inviting the therapist to share their experiential position. In recipient actions, therapists could use a combination of zero and active person reference which served to communicate an empathic stance and an invitation to the client to take an agentic observer position. Almost exclusively, only therapists used changes in footing. This could happen rapidly within single utterances and serve to express affiliation with the client's emotional experience and to invite or challenge the client to take an observer position. The study supplemented the CA change model with the DA and DSA notions of changes in agency positions as core elements in therapy talk and showed how variations in person references and changes in footing had a decisive influence on how different types of turns functioned within the overall conversational structure of the psychotherapy institution.

## 1. Introduction

According to Peräkylä (2019), conversation analysis (CA) can contribute in two ways to the understanding of how psychotherapy as a helping institution works. First, CA shows how the typical sequential organization of psychotherapeutic interaction is outlined. Second, CA can depict how the psychotherapeutic process, the realization of psychotherapeutic projects, occurs through those sequential structures. The recurring sequences involve basically a target action, i.e., any conversational entity under scrutiny, prior actions which make the target action relevant, (the therapist's) initiatory actions, (the client's) response, and finally a response to the response, the so-called (therapist's) third position action. It is through such repeated sequences that the fundamental psychotherapeutic process, Peräkylä (2019; p. 266) calls “the experience-under-transformation in psychotherapy interaction”, takes place. In the CA model, this transformation is considered to happen in three overlapping realms—referents, emotions, and relations.

This study investigated how clients and therapists, when discussing clients' problematic experiences, made use of two specific conversational practices, i.e., different grammatical forms of person reference and changes in footing, and what the consequences of these were for how the client was positioned in relation to that experience, with a specific interest in how the client was ascribed agentic or non-agentic positions. Following the CA approach (Peräkylä et al., 2008; Peräkylä, 2019), the uses of person references and changes in footing in therapists' initiative turns, clients' responses, and therapists' third position (recipient) actions (formulations, extensions, and reinterpretative statements) were examined.

Looking at person references and changes in footing, the present study seeks to show how those conversational practices contribute to the ascription of agency positions in the conversational sequences identified by CA. Person reference and footing are both related to the ascription of perspectives: in the case of person reference ascription to actor role and in the case of footing ascription to a source. A point of departure for the present study is the observation that the therapist's response, the third position action, involves a double function, i.e., on one hand showing an empathic understanding of the client's problematic experience, while on the other hand offering new positions in respect to it (Etelämäki et al., 2021). To do this, the therapist needs to communicate his/her appreciation of the client's emotional position toward the experience, and then also challenge whatever non-agentic position the client takes toward it. At best, the combination of these communicative actions affords the therapist and client to jointly construct a shared observational position (Leiman, 2012) with respect to the issues at hand.

### 1.1. Agency positioning in psychotherapy

Restoring clients' disturbed sense of agency has, from different theoretical and methodological perspectives, been seen as a core goal of psychotherapy (Wahlström, 2006a; Williams and Levitt, 2007; Mackrill, 2009; Adler, 2012; Wahlström and Seilonen, 2016; Gorlin and Békés, 2021). Two generic models of psychotherapeutic change processes, the Assimilation of Problematic Experiences Sequence (Stiles, 2001, 2011; APES) and the Innovative Moments Coding System (IMCS; Gonçalves et al., 2011), present clients as entering therapy somehow restricted in their capacity to act. APES attributes this incapacity to experiences that are psychologically avoided or unclearly defined, subjugated to dominant voices, and yet not integrated to previous experiences. IMCS presents clients as initially restricted by a problem-saturated self-narrative. The models suggest that the therapeutic process helps the client to move from a non-agentic position, bound by a silenced problematic experience or immersed in a problematic narrative, to an agentic one (Toivonen et al., 2020).

From the perspectives of discourse analysis (DA) and dialogical sequence analysis (DSA), again, the essential task in psychotherapeutic conversations is seen as to afford the client new positions in relation to his/her problematic experiences (Avdi and Georgaca, 2007; Leiman, 2012). In DSA, the client is pictured as being in an object position where he/she feels beleaguered by a problem or acted upon by it (Leiman, 2012). During the course of therapy, the object position is supposed to evolve, assisted by a mediating process where the client adopts an observer position, into an altered, empowered relationship to the problem, namely, a subject position.

Positioning is a discursive process where speakers situate themselves and others in different ways with regard to their experiences, relations, and life situations (Davies and Harré, 1990; Kurri and Wahlström, 2007; Avdi, 2012; Wahlström, 2016). In psychotherapeutic conversations, specifically, clients position themselves and are positioned by the therapists with respect to presented problems, relationships with close others, and their own “self”—their own understanding of their actions and their ways of portraying themselves. Therapeutic change, then, as it appears in sequences of interaction, can be depicted as changes in discursive positions, evolving throughout the therapy process (Kurri and Wahlström, 2005, 2007; Suoninen and Wahlström, 2009; Avdi, 2012, 2016; Deppermann et al., 2020).

Toivonen et al. (2019) have shown how discursive positions can be agentic or non-agentic. A non-agentic position entails a client's expression that he/she does not initiate actions he/she wishes to or is expected to assume or undertakes actions that are unwished for or not expected (Wahlström, 2006b). Such an expressed stance of limited action possibilities constitutes the discursive display of loss of one's sense of agency, the non-agentic self-ascription. When taking an agentic position, again, the speaker ascribes to himself or herself an active and responsible stance.

Ascriptions of agency and non-agency positions can be self-ascriptions (the client ascribes agency or non-agency to him- or herself) or other ascriptions (the therapist ascribes agency or non-agency to the client) (Toivonen et al., 2019). The ascriptions are usually not conscious or intentional discursive deeds. Rather they are side products of the participants' discursive navigation within the institution of psychotherapy. The ascriptions are thus talked into being moment by moment in the therapy conversation.

Noteworthily, recent approaches to the research on human agency (Enfield and Kockelman, 2017) accentuate its quality not only—and perhaps not even primarily—of an individual capacity but as distributed between actors. When looking into the relations between key elements of agency, such as intentionality, causality, flexibility, and accountability, it is asked how such relations are distributed among individuals, and also across other entities, for instance, bodies, minds, things, spaces, and times. The distributed agency is approached as embedded in a variety of human-specific modes of shared action, from causality, intentionality, and personhood to ethics, punishment, and accountability.

### 1.2. Person reference and change in footing in therapy talk

The use of different forms of person references in psychotherapy talk has been shown to contribute to the ascription of agency positions either to the client or other instances, or to the avoidance of agentic positions (Kurri and Wahlström, 2007; Toivonen et al., 2019; Etelämäki et al., 2021). Analyzing person reference, this study looks at the syntactic subject of clauses, not at the person in the object position. What is examined is whether the syntactic subject is filled by naming a specific person, by only implicitly referring to a specific person using an impersonal expression, or by a non-person entity, like for instance an emotion.

An interesting case in this respect is Finnish, since in addition to the first, second, and third-person singular and plural forms, it features a personal passive and a so-called zero person. The zero-person construction has no overt subject, and the predicate verb appears in the third-person singular form (Laitinen, 1995).

Active: *niissä tilanteissa hän nauraa/in those situations she laughs*.

Passive: *niissä tilanteissa nauretaan/in those situations one laughs*.

Zero: *niissä tilanteissa nauraa*.

Literally, the zero construction translates into English as ^*^ *in those situations laughs*. In this presentation, the notation Ø will be used for the zero person: *in those situations Ø laughs*.

The Finnish zero person is different from the use of an impersonal subject or the use of the generic “you” as syntactical subject of sentences in English, since, as the term implies, no syntactic subject appears in the clause. It could be looked at as one form of ellipsis (leaving out the syntactic subject like in “just hit him” instead of “I just hit him”), although no mention of this is found in the literature (Laitinen, 1995). Habermas (2006) has shown how such elliptic expressions in autobiographical narratives render an impression of the speaker's avoidance of a responsible agentic position.

Using the zero, the speaker creates as if an open space for an undefined actor or experiencer (Etelämäki et al., 2021; Suomalainen et al., 2023). When used by clients, the zero has been seen as a means to take a weak agentic position and reduce one's responsibility for problematic or untoward action (Kurri and Wahlström, 2007; Toivonen et al., 2019). When used by therapists, the zero has been seen as a means to take an empathic position toward the client's problematic experience or to sensitively invite the client to take an observer position with respect to his/her own experience or action (Etelämäki et al., 2021). In the present study, it will be shown how, in dialogue, clients and therapists can use zero as a means to create an impression of a shared agency or experience.

In conversations, interlocutors express their utterances from some point of view. This was by Goffman (1971, 1979) coined footing. Briefly defined, the footing of an expression delineates in whose interest (the principal), with whose words (the author), and with whose voice (the animator) what is said is said. Changing footing speakers display various degrees of distance from or closeness to what they are reporting. According to Goffman (1981, 128) “a change of footing implies a change in the alignment we take up to ourselves and to the others present as expressed in the way we manage the production and reception of an utterance”[SIC]. The linguistic means used for change in footing is mainly quoting someone and thereby attributing a statement to someone other than the speaker, or in the case of self-quoting to oneself from an observer's perspective.

When formulating his or her utterances, a speaker can take up the different roles of production of talk—the principal whose position the talk is meant to represent, the author who does the scripting, and the animator who is the speaker of the words—in various ways and this has different implications for the accountability of him or her (Potter, 1996). Thus, changing the participation framework of the conversation (Goodwin, 2007), the speaker, when reporting or commenting on an event, not only reports the perceived locus of causality but also the locus from which the reporting or commenting is being done. As a consequence of this, persons' rights, obligations, and possibilities to act change with the floating variance of footing. In the present study, it will be shown how changes in footing are used by therapists to modify their stance of either closeness or distance to the clients' expressions, thus taking up different positions of alignment and affiliation (Steensig, 2013).

The CA change model offers a comprehensive account of the overall conversational structure of the psychotherapy institution. The DA and DSA notions of psychotherapeutic change, again, highlight changes in the client's agency positions as core elements in therapy talk. The aim of this study was to show how variations in person references and changes in footing had a decisive influence on how different types of turns, as identified by the CA model, functioned in naturally occurring therapy conversations with respect to how the client was ascribed to different agentic positions.

## 2. Materials and methods

This study used a data corpus from nine individual psychotherapies, conducted by five therapists, that took place at a university training clinic in Finland. The sessions were conducted in Finnish. Videotaping and the use of the sessions for research purposes took place with the informed and documented consent of clients and therapists. From all therapies, the first sessions were completely transcribed and constitute the database of this study. A verbatim transcription was considered to be sufficient for the study. In this study, data extracts are shown both in the Finnish original and translated into English.

Four (all female) of the therapists were licensed psychologists, with at least 2 years of clinical practice (but usually more), who participated in a specialization program in integrative psychotherapy. One therapist was an experienced male psychotherapy trainer, who was conducting the session with one female trainee as co-therapist. Eight of the clients were female and one was male. The age range of the clients was from 19 to 45. They were all self-referred, and their presenting problems included depression, fatigue, social anxiety, stress, panic attacks, coping with divorce, and binging and purging.

Episodes, where clients and therapists discussed clients' presenting problems, were identified in the data corpus. Following a conversation analytical approach, the uses of person references and changes in footing in therapists' initiative turns, clients' responses, and therapists' third position (recipient) actions (formulations, extensions, and reinterpretative statements) were examined.

## 3. Results

### 3.1. Variations in person references

In this section, five extracts from the data are shown, exemplifying the corollaries of different uses of person references in therapeutic dialogues for agency positioning. In Extract 1, the therapist, in his initiating turn, uses active second-person reference inviting the client to observe her inner experience, but the client responds with a zero construction and an externalization of the agency.

Extract 1: Mitigating and distributing agency

01 T: if you pursue that situation in your mind, so what were you afraid of
*jos sä sitä tavottelet mielessä sitä tilannetta että mitä sä pelkäsit*
02 what was the feeling what did it tell you
*että mikä se tunne oli että mitä se kerto sulle*
03 C: well really something like that ø cannot control oneself
*no sitä ois oikeestaa että varmaa jotai että ei pysty hallitsee itteesä*
04 that ø just trembles awfully then ø freaked then all I reckon I felt
*sillai että tärisee vaa kauheesti sit säikähti sitä sillon iha että varmaa must tuntu*
05 that the bo- like when I in the seventh grade started to drink coffee
*että se ru- niinku ku mä seittemännellä luokalla alotin juomaan kahvia*
06 and my body reacted just terribly easily to something like that
*ja mun ruumis reagoi iha älyttömän herkästi semmoseen*

In his question (lines 01 and 02), the therapist, using the second-person singular you, positions the client as an active agent both in the session (“if you pursue”) and with reference to her problematic experience (“what were you afraid of”). He also gives her the feeling of an agentic position (“what did it tell you”). In her response, the client first (lines 03 and 04) gives an account of her experience using zero person (“Ø cannot”; “Ø just trembles”; “Ø freaked”), giving it a sense of generality. She then (lines 04 and 05), using the first person singular, assumes a more active position as observer and actor (“I felt”; “I started”), and then again (line 06) receding to a more non-agentic stance, gives the agentic force to her body (“my body reacted”).

In Extract 2, the therapist, in a preceding turn (prior action not shown), has described how socially anxious people often accommodate to the expectations they assume others have of them. The client, responding to this in a third position type of turn, uses zero to describe her experience as an anxious person. The therapist initially affiliates with this and then moves to the second person when formulating a goal for change.

Extract 2: Affiliating and encouraging

01 C: yeah on the other hand it's a little bit kind of one type of talent
*joo kyl toisaaltahan se on vähä niiku lahjakkuuden lajiki sitte omalla tavallaan*
02 that ø knows how, well it depends on how ø uses it, does it have any other use than
*että osaa no miten sitä sitten käyttää et onks sille mitään muuta käyttöö ku se*
03 that ø self gets even more anxious
*että ahdistuu ite entistä enemmän*
04 T: well yeas of course at that level ø can always say that when ø gets anxious then it is
*niinpä et tietenki siin vaiheessa voi aina sanoo että ku ahdistuu ni sithän se on*
05 that kind of too extreme, of course it is also a kind of social skill that ø knows how
*semmosta liiallista et tokihan se on semmosta sosiaalista taitookin et osaa*
06 an important skill, but so that it wouldn't happen at the expense of oneself
*ihan tärkee taito mutta ettei se tulis niinku itsen kustannuksella*
07 in your case I would see […] that you would be so much at turns with yourself
*et kyl mä näkisin niinku sun kanssa […] et pääsisit itses kanssa sen verran sinuiks*
08 that you would dare to be yourself in those situations
*että et uskaltaisit niiku olla omana itsenä niissä tilanteissa*

In her turn (lines 01 and 03), the client, using zero person (“Ø knows”; “Ø uses”; “Ø gets”) in a generalizing way of speaking, ponders the pros and cons of such an inclination, naming it “a type of talent”. The therapist responds to this (lines 04 and 05), continuing the use of zero person (“Ø can”; “Ø gets”; “Ø knows”), by first joining the client's point of view but then (lines 07 and 08), when proceeding to pose a target for change for the client, changes in the use of an active person reference (“I would see”; “you would be”; “you would dare”).

Before Extract 3, in a preceding turn (prior action not shown), the client has described how she has sought her father's acceptance through achievements in school and in work. In the extract, she tells how her sister was quite different and what the impact of this was on her. In her turn, the client moves from using the active first person when referring to her self-positioning in childhood to zero when pondering its effects on her conduct in adulthood. The therapist responds with an interpretative formulation using second-person reference.

Extract 3: Co-constructing an interpretation

01 C: and then as my older sister again is that kind of a strong and crackling person
*ja sitte kun mun isosisko on semmonen taas voimakas räiskähtelevä persoona*
02 then it was she who objected and slammed doors and was snappy then I felt
*ni se oli niinku se joka pisti hanttiin ja paisko ovia ja kiukutteli ni sitte must tuntu*
03 that I even less dared to lift my head up when I saw that I did not want
*et mä vielä vähemmän niiku uskalsin nostaa sieltä päätäni että ku mä näin että mä en halua*
04 those kind of quarrels so ø kind of conformed and ø conceded
*tommosia kahnauksia että sitä sit niinku sopeutu ja jousti*
05 and ø always took the chores that my sister left undone and probably
*että otti aina ne hommat mitä siskolta jäi ja silleen*
06 somehow ø adapted the role of a nice girl quite strongly
*jotenki semmosen kiltin tytön roolin omaksunu varmaa aika vahvasti*
07 t: mm have you now then by falling ill with not-doing by revolting here then
*mm oot sää nyt sitten sairastumalla eisuorittamiseen ni kapinoimalla tässä nyt sitte*
08 like i do not want to be like this anymore

*että mä en enää halua tällainen olla*

When she refers to what happened in the past (lines 01 and 03), the client uses active person reference (“I felt”; “I dared”; “I didn't want”). When she then in the latter part of the turn (lines 04 and 06), on a more general note, describes the impact of this relational setting on her personal dispositions she resorts to the use of the zero construction (“Ø conformed”; “Ø conceded”; “Ø took”; “Ø adapted”). The therapist's response (lines 07 and 08), using the metaphor “falling ill with not-doing”, is a challenging and interpretative formulation of the client's initial presenting problem of not being able to perform professionally and privately as before. The therapist gives force to this (re)formulation by using active person reference (“have you now”; “I don't want”), introducing the word “revolt”, and changing the footing of speech (line 08), using the client's voice (“I don't want to be like this anymore”).

In Extract 4, the therapist, in her third position turn, offers a formulation of the client's present problematic situation in life and a desirable way of action. When doing so, she makes use of plenty of discursive means to mitigate her own agency position and any allusion that her suggestion could be seen as a demand or challenge.

Extract 4: Offering a solution delicately

01 T: that is what I also actually listen to that there has been an awful lot of things
*sitä mäki tässä oikeestaan niinku kuuntelen että et hirveen paljon ollu niitä asioita*
02 somehow in a short time and somehow that it comes like that kind of a feeling
*tavallaan lyhyessä ajassa ja ja tota jotenki se että et se tulee niinku semmonen tunne*
03 right that that y- kind of you yourself said s- defined somehow that you would wish
*justiin että että s- niinku sä itekki sitä sanoit s- määrittelit jotenki niin että et sä toivoisit*
04 that everything somehow would become clear and would be somehow solved that
*että kaikki jotenki kirkastuis ja olis jotenki selvää et*
05 somehow the whish that that ø could somehow like make some kind of decision on
*jotenki se toive siitä että että pystyis jotenki niinku tekemään jonkinlaisen päätöksen siitä*
06 what direction ø now really is like going
*et mihin suuntaan nyt tosiaan on niinku menossa*

The therapist's turn is loaded with delicacy markers. She uses frequently the expressions “somehow” and “kind of”, repeats words and seems to hesitate in choosing words and avoids giving an impression of taking a strong personal stand. By using the expressions “I also” (line 01) and “you yourself” (line 03), the therapist constructs a shared agency position with the client. Moreover, she mitigates her own agency by attributing agentic force to the impersonal “feeling” (line 02 “it comes like that kind of a feeling”). The active “you” in the formulation (lines 03 and 04) is softened to a zero construction (line 05 “that Ø could … make some kind of decision”) in the offering of the potential solution to the client's predicament.

Extract 5 shows another instance where the therapist mitigates her own agentic position in favor of strengthening that of the client's. The therapist offers a rephrasing formulation, aiming at giving additional force and partly new meaning to the client's expression of a wish for change given earlier in the conversation. In the therapist's turn, the actual formulation is given a rather elaborate ground.

Extract 5: Rephrasing and strengthening a wish for change

01 T: yeah somehow it co- comes such a feeling that even if now there is no alcohol
*nii jotenki tu- tulee semmonen tunne että et siitä huolimatta et vaikka nyt ei oo alkoholia*
02 or drugs otherwise involved even then it somehow sounds as if you were
*eikä eikä päihteitä muuten mukana niin tavallaan siis kuulostaa siltä niinku sä*
03 afraid somehow of being going back to something of the same as before
*pelkäisit jotenki sitä että että sä oot menossa johonki semmoseen samaan mihin aikasemmin*
04 C: yeah
*joo*
05 T: that the same kind of treadwheel just wants to go on
*et se sama ikäänku oravanpyörä (2.0) tahtoo aina vaan pyöriä*
06 C: yes
*kyllä*
07 T: so you would want to get somehow off it
*ni sä haluaisit siitä jotenki pois*

As in Extract 4, this same therapist, here with another client, uses at the beginning of her turn (line 01) the phrase “it comes such a feeling”, thus mitigating her agentic position as the author of the statement to come. The expression is further softened by referring both to the client's problematic behavior (lines 01 and 02 “now there is no alcohol or drugs involved”) and her own stance (line 02 “it somehow sounds”) in an impersonal manner. Then, when preceding to give the actual formulation, the therapist changes to active person reference (lines 02 and 03 “as if you were afraid”). Thus, the emotion as the motivational force for change is attributed to the person of the client, while, as the formulation continues, using the treadwheel metaphor (line 05) the agency of resisting change is offered to an impersonal force. The client's minimal responses give the impression of a positive uptake.

### 3.2. Change in footing

In Extract 3, it was shown how the therapist, when delivering a rather challenging interpretative formulation (“have you now then by falling ill with not-doing by revolting”), at the end of her turn swiftly changed the footing of her talk by animating the client's supposed private thought (“I don't want to be like this anymore”). In the present data, such rapid and brief changes in footing appeared fairly frequently as discursive means used by the therapists. They were usually utilized, as in Extract 3, as a part of a formulation and served, by presenting the therapist as having access to the client's experience and sharing the client's position, with the intent to make the formulation more appealing to the client.

In Extract 6, the therapist first uses change in footing when wording a rephrasing formulation as part of a specifying question. Later she gives another formulation, again using change in footing, now serving to open a possibility to extend the scope of the conversation. The client has gone through a divorce and sought therapy due to her difficulty to let go of her feelings for her ex-husband. Earlier in the conversation (prior action not shown), she has pondered whether her feelings are “her true own feelings” or if she is only selfishly manipulating others. Now in her turn, the therapist explores whether this negative self-concept is due to the divorce or a more long-lasting experience.

Extract 6: Exploring the client's experience and broadening the scope of discussion

01 T: has this kind of experience that that somehow that I am not good or that
*onks tää tämmönen kokemus siitä että et et jotenkin et mä en ookkaan hyvä tai et*
02 I would be somehow selfish then have you had that kind of already earlier
*mä olisin jotenki itsekäs ni onks sulla ollu sellasta jo aiemmin*
03 or is it something that has now with the divorce kind of come up
*vai onks se semmonen asia mikä on nyt eron myötä niinku noussu*
04 C: it has been earlier too quite sure but
*on sitä ollu aiemminki ihan ihan varmasti joo mut*
05 T: what it really is how I am
*mitä se oikeesti on millanen mä oon*

In her question (lines 01 and 02), the therapist animates the client's thought (“I am not good”; “I would be somehow selfish”), using change in footing to give a rephrasing formulation. The client's response (line 04) is affirmative, still including the qualifying “but”. Disregarding this, the therapist continues (line 05) with another formulation in a rather challenging way (“what it really is how I am”), thus offering a broader topic, the client's self-understanding at large, to be discussed.

Typically, as in Extract 6 and usually, in the present data, change in footing appeared as part of different types of therapists' third position turns. Extract 7 shows a quite untypical case where the client changes footing when quoting her own inner dialogue and the therapist follows suit in her response. The client is reflecting on a new understanding of how her problematic experience in group situations develops.

Extract 7: Aligning, affiliating, extending, and interpreting

01 C: does it go like this that when i get anxious and there comes those physical symptoms
*meneekö se nyt niin että kun minua jännittää ja siihen tulee niitä fyysisiä oireita*
02 then i start to be one hundred times more anxious that now I' m there somewhere
*niin minua alkaa jännittää sata kertaa enemmän että nyt olen jossain tuolla*
03 in front talking that this won't work that I'll get lost of breath and then
*edessä puhumassa että tästä ei tule mitään että mulla loppuu hengitys ja sitten*
04 from that comes that kind of terrible panic that I won't make it that I can't handle
*siitä tulee semmoinen kauhea paniikki että mä en selviä että mä en pysty*c
05 this that if i can't then this job will suck
*tähän että jos mä en pysty niin tämä homma menee pilalle*
06 that I haven't been able to do my own
*että en ole pystynyt hoitamaan omiani*
07 T: now you start to describe the inner process what starts to happen in your mind
*nyt sinä alat kuvaamaan sisäistä prosessia että mitä sinun mielessä alkaa tapahtua*
08 when you are, for instance, in some seminar and it is your turn to present your own work
*kun sä oot esimerkiksi jossain seminaarissa ja sinulla on oma vuoro esittää sitä omaa työtä*
09 then you start to notice physical symptoms and you start to have those kind of thoughts
*niin sä alat huomaamaan fyysisiä oireita ja sinulle alkaa tulla tuon tyyppisiä ajatuksia*
10 in your mind that what now how do I survive this and if I don't survive then terrible
*mieleen että mitäs nyt sitten miten mä selviän tästä ja jos mä en selviä niin kauheata*
11 then this will totally suck
*niin sitten tämä menee ihan pilalle*

At the beginning of her turn (lines 01 and 02), the client ponders on how her anxiousness rises when she notices her physical reactions, and then in line 03, she, in the form of a self-quotation, animates her own inner dialogue (“this won't work”). Furthermore, in lines 04 and 05, she uses the same kind of change in footing (“I can't handle this … if I can't then this job will suck”) to enliven the psychological cumulation of her panicking experience. The therapist responds with a rather elaborated third position turn, designed partly as an extension of the client's account (line 07 “you … describe the inner process”; line 08 “you are for instance in some seminar and it is your turn to present your own work”) and partly as an interpretation linking the physical experience to the psychological one (line 09 “you start to notice physical symptoms and you start to have those kind of thoughts”). She finishes the turn with a change in footing quoting the supposed thought (lines 10 and 11 “what now how do I survive this and if I don't survive then terrible then this will totally suck”). With the use of the word “mind” (lines 07 and 10), the therapist constructs a shared object for inspection with its own agentic position in the formation of the client's problematic experience—an experience to which both client and therapist can have access.

## 4. Discussion

The CA model of psychotherapeutic conversational order and change process, as presented by Peräkylä (2019), delineates a specific sequential organization and an outline of how transformations of the client's experience with regard to issues (referents), emotions, and relations are realized through the typified conversational sequences. The therapist's so-called third position actions, i.e., responses to the client's expositions of his/her problematic experiences, are given particular attention in the model.

The aim of the present study was 2-fold. First, to supplement the CA change model with the DA and DSA notions of changes in agency positions as core elements in therapy talk, and, second, to show how therapists and clients in dialogue made use of variations in person references and changes in footing as discursive means to handle subtle modifications of agency ascriptions. It was shown that such changes in discursive practices had a decisive influence on how different types of turns, as identified by the CA model, functioned within the overall structure of the psychotherapy institution.

The analysis showed that in initiative turns, for instance, questions, therapists usually used the second-person singular, which marked the turn as an invitation for the client to respond from his/her personal point of view, thus ascribing active agency to the client. When telling their problematic experiences, clients typically used the so-called zero-person constructions, a particular grammatical form of person reference in spoken Finnish. This form of expression functioned to present the client's experience as common to people in general. Such a presentation, again, served both to lessen the client's agency and invite the therapist to share the client's experiential position. In recipient actions, such as formulations, extensions, and reinterpretative statements, therapists could use a combination of zero and active person reference which served to communicate, on one hand, an empathic stance and, on the other hand, an invitation to the client to take an agentic observer position.

Almost exclusively, only therapists used changes in footing. This could happen rapidly within single utterances and served to express affiliation with the client's emotional experience—when changing footing to the client as principal and animator—and to invite or challenge the client to take an observer position—when changing back to self as principal and animator. Change in footing, as used by clients, was rare. When occurring, it usually had the form of self-quotations and served to animate the client's private dialogue, thus helping the speaker to adopt an observer position with respect to his/her own emotional relation to a problematic experience.

By showing how therapists and clients apply subtle variations in language use, this study contributes to a large body of discursively oriented research on the actual accomplishment of therapeutic actions (Strong and Smoliak, 2018). Interactional research has explicated the particularities of how clients and therapists in conversation perform, among others, empathic understanding (Voutilainen, 2012; Weiste and Peräkylä, 2014), building of working alliance (Muntigl et al., 2012; Muntigl and Horvath, 2014), challenging of beliefs (Weiste et al., 2016), and production of new meanings (Vehviläinen, 2003; Kykyri et al., 2017). In the present data, therapists conveyed empathic understanding by echoing clients' impersonal expressions, built working alliances by shifting rapidly between zero and active person reference, challenged beliefs with interpretative formulations, and used changes in footing to animate new meanings in clients' private dialogue under transformation.

One particular point of view of the present study was to show how therapists, using the observed microscale changes in linguistic expression, achieved therapeutic responsiveness (Stiles et al., 1998; Leiman and Stiles, 2001; Penttinen et al., 2017) toward clients' self-positionings and emotional expressions, as these were embedded in their presentations of their problematic experiences. Such responsiveness could also pave the way for possible new agency positionings. The analysis opens up a new perspective on therapist responsiveness, looking at it as the discursive achievement of distributing and sharing experience, as well as an agency (Etelämäki et al., 2021), between the interlocutors. This point of view could also be adopted in the study of how operating within the client's so-called therapeutic zone of proximal development (Leiman and Stiles, 2001) is performed.

The findings of the study are mainly reported as different incidences of person references and changes in footing, and their connections to ascriptions of agentic or non-agentic positions. Some of the claims made, like the suggestion in Extract 2 that the illustrated conversational interaction, suggests a pattern of “affiliating and encouraging” should preferably have been backed up by a more extended sequential analysis. Space being restricted, the intention is not to claim that it is merely the choice of personal preference and/or footing which accomplishes an affiliative interaction between the therapist and the client. The reporting of the findings as a somehow fragmented picture of various, different patterns without a coherent, connecting thread, suggestive of the broader picture, may undoubtedly be seen as a limitation of the study. Then, on the other hand, this may also reflect the genuine character of therapeutic dialogues, as represented in the current data.

Evidently, the variations in the uses of linguistic expressions shown in the data were rather spontaneous than deliberate. They appeared, in speech acts by clients and therapists alike, as natural utilizations of linguistic resources. Even then were the consequences of these variants of conversational practices for agency ascriptions and therapeutic collaboration significant. From the point of view of clinical relevance, the question arises whether such diversity in linguistic performance could be incorporated deliberately into therapist skills and repertoires. Leaving that question open, this study can contribute to clinical practice by making therapists more sensitive toward the meaningfulness of even small nuances in linguistic presentations.

The zero-person reference as a grammatical construction is unique to Finnish and commonly used in spoken language. This study showed how speakers in psychotherapeutic dialogue used this linguistic resource to achieve discursive and conversational ends. It is, of course, conceivable, and even plausible, that such interactional functions are operating in therapeutic conversations conducted in other languages too. Comparative studies of therapy talk in different languages should shed light on the different linguistic means toward such ends.

## Data availability statement

The raw data supporting the conclusions of this article will be made available by the authors, without undue reservation.

## Ethics statement

Ethical review and approval was not required for the study on human participants in accordance with the local legislation and institutional requirements. The patients/participants provided their written informed consent to participate in this study. Written informed consent was obtained from the individual(s) for the publication of any potentially identifiable images or data included in this article.

## Author contributions

The author confirms being the sole contributor of this work and has approved it for publication.
